# Significance of the Differential Peptidome in Multidrug-Resistant Tuberculosis

**DOI:** 10.1155/2019/5653424

**Published:** 2019-01-17

**Authors:** Yan Yang, Jianqing Wu

**Affiliations:** ^1^Department of Geriatrics, First Affiliated Hospital, Nanjing Medical University, Nanjing, Jiangsu 210029, China; ^2^Department of Tuberculosis, The Second Hospital of Nanjing, Nanjing, Jiangsu 210003, China

## Abstract

Most multidrug-resistant tuberculosis (MDR-TB) patients fail to receive a timely diagnosis and treatment. Therefore, we explored the differentially expressed peptides in MDR-TB compared with drug-susceptible tuberculosis (DS-TB) patients using LC-MS/MS and Ingenuity Pathway Analysis (IPA) to analyse the potential significance of these differentially expressed peptides. A total of 301 peptides were differentially expressed between MDR-TB and DS-TB groups. Of these, 24 and 16 peptides exhibited presented high (fold change ≥ 2.0, P < 0.05) and low (fold change ≤ −2.0, P < 0.05) levels in MDR-TB. Significant canonical pathways included the prothrombin activation system, coagulation system, and complement system. In the network of differentially expressed precursor proteins, lipopolysaccharide (LPS) regulates many precursor proteins, including four proteins correlated with organism survival. These four important differentially expressed proteins are prothrombin (F2), complement receptor type 2 (CR2), collagen alpha-2(V) chain (COL5A2), and inter-alpha-trypsin inhibitor heavy chain H4 (ITIH4). After addition of CR2 peptide, IL-6 mRNA expression in THP-1 cells decreased significantly in dose- and time-dependent manners. Cumulatively, our study proposes potential biomarkers for MDR-TB diagnosis and enables a better understanding of the pathogenesis of MDR-TB. The functions of differentially expressed peptides, especially CR2, in MDR-TB require further investigation.

## 1. Introduction

Tuberculosis (TB), which is caused by* Mycobacterium tuberculosis* infection, is a potentially fatal disease. According to the World Health Organization (WHO) report, the number of active TB patients in 2015 exceeded 20 million worldwide, and there were 9.6 million newly diagnosed TB cases and 1.5 million cases of mortality [1]. The emergence of drug-resistant tuberculosis is a serious threat to global public health security, substantially increasing the burden of global tuberculosis control. Globally, 3.5% of new and 20.5% of previously treated TB cases are multidrug-resistant tuberculosis (MDR-TB), with 210,000 cases of mortality. The number of drug-resistant tuberculosis patients in China accounts for approximately 20% of the worldwide cases, and 11.6% of new, and 35.9% of previously treated TB cases have MDR-TB. Only 48% of MDR-TB patients are successfully treated [1].

In the treatment of drug-sensitive TB, the abuse of anti-TB drugs, irrational treatment programmes, and inadequate drug administration are the most common reasons that cause MDR-TB. Moreover, most MDR-TB patients fail to obtain a timely diagnosis: only 19% of MDR-TB cases worldwide are diagnosed, whereas less than 10% of MDR-TB patients in China are diagnosed [2]. Therefore, the key to the control of MDR-TB is early diagnosis. Diagnostic methods for MDR-TB include phenotypic and molecular diagnostic techniques. Drug susceptibility testing (DST) is the golden standard for MDR-TB diagnosis, and it is also an important basis for the formulation of MDR-TB treatment programmes [3]. However, the DST method is time-consuming, and the detection rate is low; thus, it is not suitable for early diagnosis and treatment. Microscopic-observation drug susceptibility (MODS) testing shortens the time of tuberculosis culture to 1-2 weeks [4]; however, false positives are likely. Results using the fluorophage method are rapidly obtained, though the technique is expensive, and matched 100% with the results of DST of first-line anti-TB drugs [5]. Regardless, the detection of second-line anti-TB drugs requires further investigation. The polymerase chain reaction-restriction fragment length polymorphism (PCR-RFLP) method only detects drug resistance-related gene mutations [6, 7], yet mutation of the targeted gene does not fully explain all mechanisms of MDR-TB [8]. Therefore, we urgently need novel biomarkers for the rapid and convenient diagnosis of MDR-TB.

Proteomic analysis may represent a valuable tool in the advancing search for biomarkers of MDR-TB. Manju Lata et al. [9] identified 14 proteins with increased intensities in OFX-resistant* M. tuberculosis* isolates compared to susceptible isolates. Wang et al. [10] reported 50 proteins and 43 miRNAs differentially expressed in serum samples from MDR-TB patients and established the MDR-TB diagnostic model based on five biomarkers. Thus, understanding the protein composition may advance understanding of the mechanisms of MDR-TB and facilitate the development of a novel diagnosis of MDR-TB. In recent years, peptidomics has become an emerging branch of proteomics that targets protein fragments, referred to as endogenous peptides; it has increasingly been applied in disease research, including the screening of disease biomarkers, diagnosis, treatment, and monitoring. The low-molecular-weight proteome may uncover previously undiscovered alternatives worthy of investigation. Previous studies have demonstrated the effectiveness of peptidomic methods. Villanueva et al. determined that a specific signature of serum peptides was able to distinguish patients with 3 different types of solid tumours from individuals without cancer [11]. More recently, Anand Bery et al. [12] identified and catalogued over 777 peptides from ovarian cancer ascites and determined that these fragments were derived from the proteins vitronectin, transketolase and haptoglobin. The authors speculated that peptidomics can be used to identify previously undiscovered disease-specific endogenous peptides that warrant further investigation as biomarkers for ovarian cancer. However, to date, the peptidomics approach has not been reported in the field of MDR-TB. In this study, we investigated serum peptide profiles from MDR-TB patients using liquid chromatography-tandem mass spectrometry (LC-MS/MS) and Ingenuity Pathway Analysis (IPA) to determine the relevance of unique endogenous peptides and to explore bioactive peptides in the pathogenesis of MDR-TB.

## 2. Materials and Methods

### 2.1. Sample Collection and Processing

**Figure 1:**
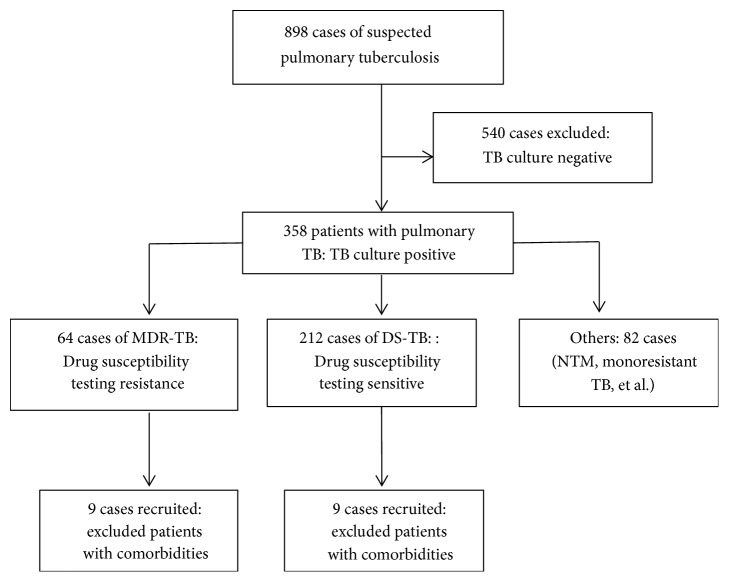
Flow diagram for the selection of the participating patients, according to the STROBE guidelines.

Blood samples were obtained from 18 pulmonary tuberculosis patients who were enrolled in this study and provided written informed consent from March 2016 to December 2017 (Figure 1 and Table 1). Nine patients were diagnosed with MDR-TB (defined as TB strains resistant to at least isoniazid and rifampin) by DST, and their ages ranged between 21 and 52 years old (median 33.2). The remaining 9 patients were drug-susceptible tuberculosis (DS-TB) patients, and their ages ranged between 15 and 65 years old (median 30.2). No subject had a prior history of TB treatment or suffered from other complications. There was no significant difference between the groups in smoking or alcohol abuse. The research protocol was approved by the Ethics Committee for Human Research, First Affiliated Hospital, Nanjing Medical University.

The MDR-TB and DS-TB patients were randomly divided into three subgroups, and each subgroup consisted of a mixture of 3 ml of plasma (1 ml from every 3 patients). Following blood collection, the serum samples were centrifuged at 4000 rpm for 10 min at 4°C within 2 h of collection, aliquoted with protease inhibitor (Complete mini EDTA-free, Roche Applied Science, Indianapolis, IN, USA) and stored at -80°C until analysis. The protein concentrations in all samples were determined by the bicinchoninic acid method (Pierce) using BSA as a standard.

### 2.2. Sample Treatment and LC-MS/MS Analysis

Serum samples were diluted in a denaturing solution (7M urea, 2M thiourea, and 20mM DTT) and transferred to filter devices for centrifugation. The method was confirmed by mass spectrum analysis, which can identify more peptides than previous studies on endogenous peptides [13–15]. According to the manufacturer's instructions, the blood supernatants were first ultrafiltered using a 30-kDa molecular weight cut-off filter, and the filtrates were then centrifuged using a 10-kDa filter to completely remove proteins (e.g., albumin, globulin and fibrinogen) abundant in cord plasma. The peptides in the different samples were then separately labelled in solution with isotopomeric dimethyl labels [16]. The labelled samples of the MDR-TB and control groups were mixed and simultaneously analysed by LC-MS/MS [17].

### 2.3. Bioinformatics

The LC-MS/MS data were searched using the Mascot search engine against the SwissProt sequence database with the* Homo sapiens* subset. The error tolerances were set to 15 ppm for precursor ion masses and 0.6 Da for fragment ion masses. The search results were compiled into a protein list using ProteinScape. To categorize the identified peptides, the results were analysed using the software program IPA (Ingenuity Pathway Analysis) and the UniProt Database.

### 2.4. THP-1 Cell Culture and Treatment with Peptides

To determine the effects of the F2 and CR2 peptides on the pathogenesis of MDR-TB, we treated THP-1 macrophages with F2 and CR2 peptides in vitro. Human acute monocytic leukaemia THP-1 cells were cultured in RPMI 1640 culture medium containing 10% foetal bovine serum, penicillin-streptomycin, and L-glutamine. F2 and CR2 peptides were synthesized by the company ShangHai Science Peptide Biological Technology (Shanghai, China) according to our results (F2: TFGSGEADCGLRPLF; CR2: AGLLGVFLALVA). N-terminal F2 and CR2 peptides were tagged with RKKRRQRRR-A- for acetylation to improve cell permeability. Cells were suspended in 6-well plates at a concentration of 10^6^ cells per millilitre in antibiotic-free supplemented medium and coincubated with either medium alone as a negative control, IFN-r and LPS as a positive control, or different concentrations of F2 and CR2 peptides. The cells were collected after incubation at 37°C in 5% CO_2_ for 4 h, 12 h, and 24 h for real-time PCR analysis. Two independent experiments were carried out for the treatment of peptides.

### 2.5. qPCR of Proinflammatory Factor Expression of THP-1 Cells

Total RNA was isolated using TRIzol reagent (Invitrogen, Carlsbad, California, USA) according to the manufacturer's instructions. Total mRNA (500 ng) was reverse transcribed in a 20-*μ*l reaction mixture using an iScript cDNA Synthesis Kit (Bio-Rad Laboratories, Hercules, California, USA). Quantitative real-time polymerase chain reaction (qRT-PCR) was performed using SYBR Green and optimized in the MyQ Single-Color Real-Time PCR Detection System (Bio-Rad). Gene expression was normalized to that of *β*-actin. The relative mRNA expression levels of IL-1*β*, TNF*α*, IL-6, and IL-8 were calculated using the comparative cycle threshold method [18]. All experiments were carried out in triplicate.

### 2.6. Statistical Analysis

Data from quantitative experiments were analysed using unpaired t-tests in GraphPad Prism 7.0 (GraphPadSoftware, San Diego, CA). Peptides were considered to be significantly altered between the MDR-TB and DS-TB groups at a statistical P value of less than 0.05 and a fold change of 2 or greater. All data are shown as means ± standard deviations (SDs).

## 3. Results

### 3.1. Characteristics of Peptides Identified between MDR-TB and DS-TB

**Figure 2:**
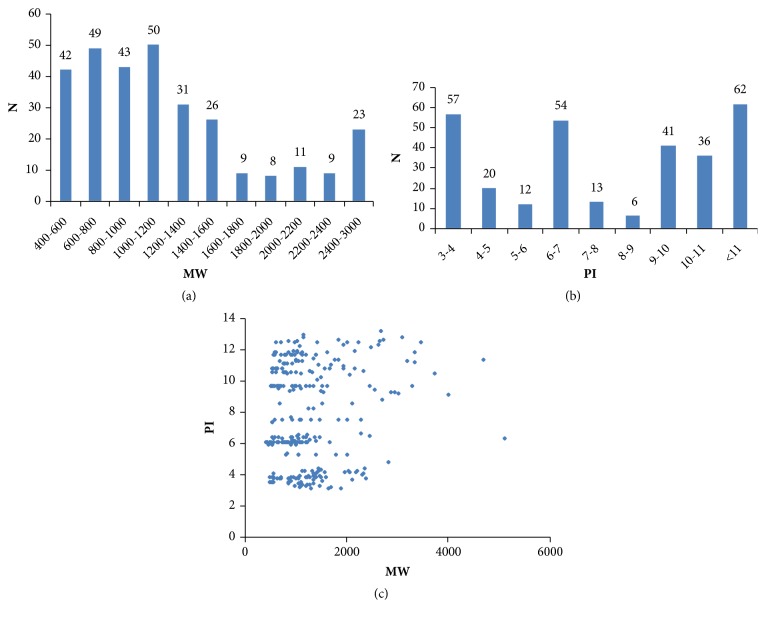
Distributions of identified differentially expressed peptides. (a) Distribution of the molecular weights (MWs) of the 301 peptides. (b) Distribution of isoelectric points (pIs) of the 301 peptides. (c) Scatter plot of MW versus pI distribution of the peptides. The data in all panels include differentially expressed peptides identified between multidrug-resistant tuberculosis and control sample groups.

The peptide compositions of pooled serum from MDR-TB were directly analysed by LC-MS after isotopomeric dimethyl labelling. A total of 301 peptides were identified as differentially expressed peptides between the MDR-TB and control sample groups (Table S1). We initially analysed the general features of the differentially expressed peptides. Both the molecular weight (MW) and isoelectric point (PI) of these peptides were distributed over wide ranges; however, most peptides were between 400-3000 Da and a PI range of 3.0–11.0, as shown in Figures 2(a) and 2(b). Moreover, the relative distribution of the PI versus the MW had a unique characteristic, whereby these identified peptides mainly gathered into three groups, which were distributed around PI 4, PI 6, and PI 10 (Figure 2(c)).

### 3.2. Cleavage Site Patterns of Identified Endogenous Peptides

**Figure 3:**
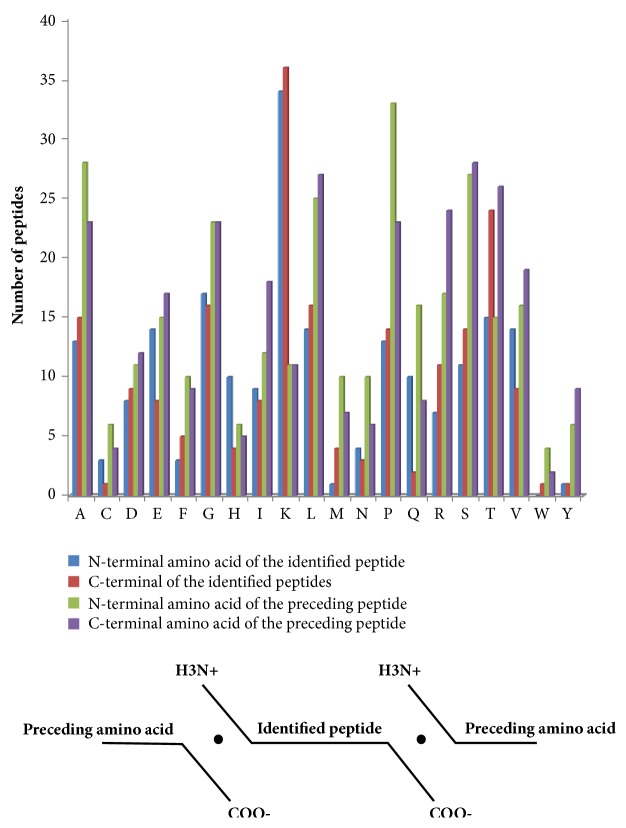
Comparison of the amino acid profiles of the four cleavage sites in each of the differentially expressed peptide fragments (S: starting amino acid of peptides, E: ending amino acid of peptides, P: previous amino acid of peptides, A: after amino acid of peptides).

LC-MS/MS identified 301 peptides, and the bioinformatic analysis showed the specificity of the four cleavage sites (Figure 3). Proline (P) was the most common amino acid at the N-terminal cut site of the preceding peptide, and lysine (K) was the most common N-terminal amino acid of the identified peptide. Serine (S) was most commonly identified at the C-terminus of the preceding peptide, and lysine (K) was most commonly identified at the C-terminus of the identified peptide. The distributions of the cut sites in the differentially expressed peptides were also variable in the samples. To further investigate the peptidome in these samples, we subsequently employed bioinformatic analyses of the cleavage sites of both the N- and C-termini (Figure 3). In general, the results of our investigation of these four cut sites reflected the findings reported in endogenous proteolytic enzymes in human serum.

### 3.3. Putative Functional Peptides

**Figure 4:**
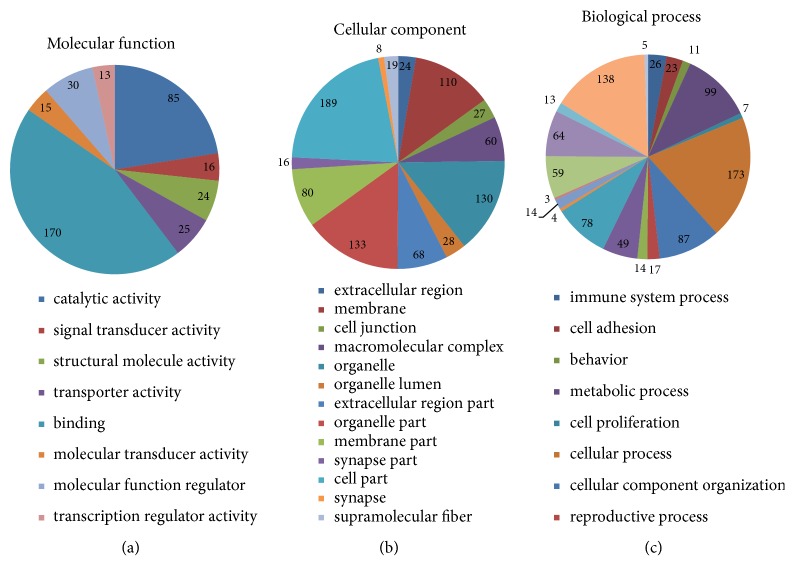
Gene ontology and homology analyses of the 301 identified peptide precursors. (a) Molecular functions of the multidrug-resistant tuberculosis peptide precursors. (b) Cellular components of the peptide precursors. (c) Biological processes of the peptide precursors.

GO analysis was performed to determine whether the precursors of the identified peptides could be attributed to particular subcellular compartments or protein classes. We categorized the subcellular locations and functions of all 301 peptides in accordance with their annotations in the UniProt database. None of the categories were considerably different, indicating that the ultrafiltration preparation method is universal in that it does not lead to preferential extraction of proteins from specific cellular compartments or with specific functions. We determined that organelle and cell parts are the predominant subcellular locations for peptide precursors. With respect to function, the majority of the proteins identified are involved in binding (45%) or catalytic activity (22%). Of particular note, the number of identified peptides with functions in the immune system process was 26 (Figure 4).

**Figure 5:**
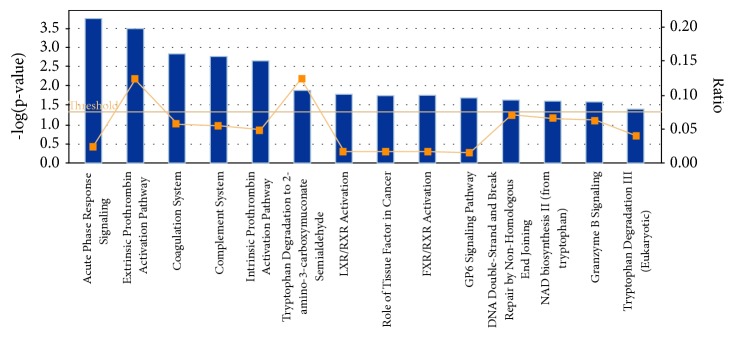
Pathway analysis was used to analyse significant canonical pathways associated with the peptide precursors that are differentially expressed in the sera of patients in the multidrug-resistant tuberculosis group.

Pathway analysis indicated significant canonical pathways associated with the peptide precursors that are differentially expressed in the serum of the MDR-TB group. Significant canonical pathways were the prothrombin activation system, coagulation system, and complement system (Figure 5).

### 3.4. Peptide Identification and Quantitative Analysis

**Figure 6:**
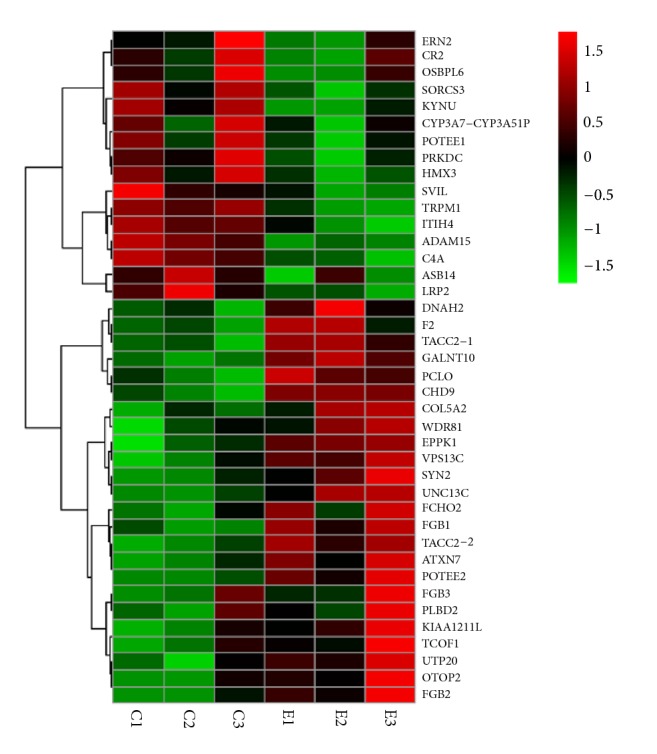
Hierarchical clustering of 40 peptides differentially expressed in multidrug-resistant tuberculosis compared with the control group.

Of 301 differentially expressed peptides between the MDR-TB and control sample groups, 24 presented high levels (a fold change ≥ 2.0, P < 0.05) and 16 low levels (a fold change ≤ −2.0, P < 0.05) in MDR-TB compared with the control group (Table 2). We also performed hierarchical clustering of 40 peptides differentially expressed in MDR-TB compared with the control group (Figure 6). Among these peptides, 3 peptides that originated from Fibrinogen *β* chain (FGB) were significantly increased in MDR-TB.

### 3.5. Networks of Differentially Expressed Precursor Proteins and Prediction Functions of Bioinformatics

**Figure 7:**
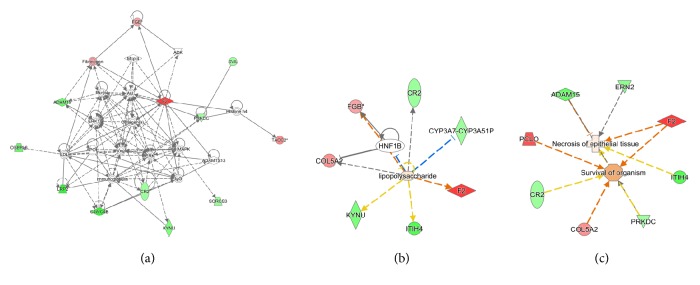
Biological functions, diseases, and networks of differentially expressed precursor proteins were analysed using the software program IPA (Ingenuity Pathway Analysis). Proteins in red are upregulated, and proteins in green are downregulated. (a) Network of differentially expressed precursor proteins. (b) LPS regulates many precursor proteins. (c) Several differentially expressed proteins were correlated with organism survival.

The biological functions, diseases, and networks of the differentially expressed precursor proteins were analysed using the software program IPA. In the network of differentially expressed precursor proteins, 13 proteins were correlated with each other or ERK, P38MAPK and AKT. LPS regulated many precursor proteins, and four proteins were correlated with organism survival. These four important precursor proteins included prothrombin (F2), complement receptor type 2 (CR2), collagen alpha-2(V) chain (COL5A2) and inter-alpha-trypsin inhibitor heavy chain H4 (ITIH4) (Figure 7).

**Figure 8:**
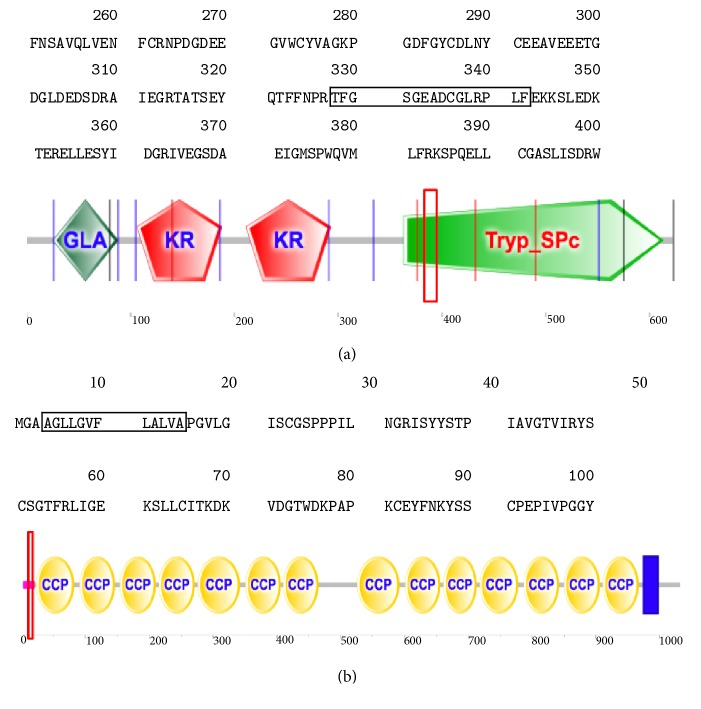
The stable functional domains of peptides. (a) TFGSGEADCGLRPLF (P00734/F2). (b) AGLLGVFLALVA (P20023/CR2).

Subsequently, we analysed the 4 important differently expressed peptides using the online tool SMART (http://smart.embl-heidelberg.de/smart/batch.pl), which indicated that 2 peptide sequences (F2 and CR2) are located in the conserved functional domains of their protein precursors. The results further showed the stable functional domains of peptides, in which TFGSGEADCGLRPLF (P00734/F2) (Figure 8(a)) and AGLLGVFLALVA (P20023/CR2) (Figure 8(b)) are located, might be closely related with drug resistance in tuberculosis.

### 3.6. Effects of F2 and CR2 on Proinflammatory Factors Expression of THP-1 Cells

**Figure 9:**
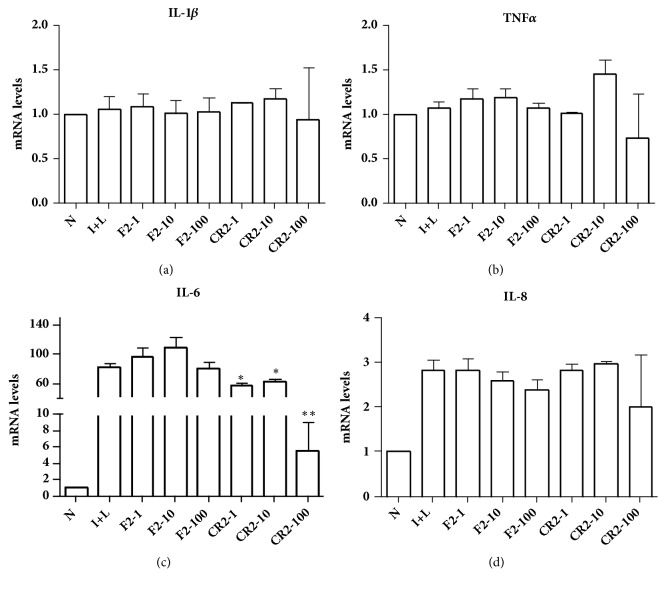
Inflammatory effects of differently expressed peptides (F2 and CR2: 1 *μ*M, 10 *μ*M, and 100 *μ*M) to THP-1 cells. (a) IL-1*β* mRNA expression did not change after the addition of F2 and CR2. (b) TNF*α* mRNA expression did not change after the addition of F2 and CR2. (c) IL-6 mRNA expression significantly decreased after the addition of CR2 (*∗*P < 0.05; *∗∗*P < 0.01), but there was no change after the addition of F2. (d) IL-8 mRNA expression did not change after the addition of F2 and CR2 (N: negative control; I+L: LPS and r-IFN).

**Figure 10:**
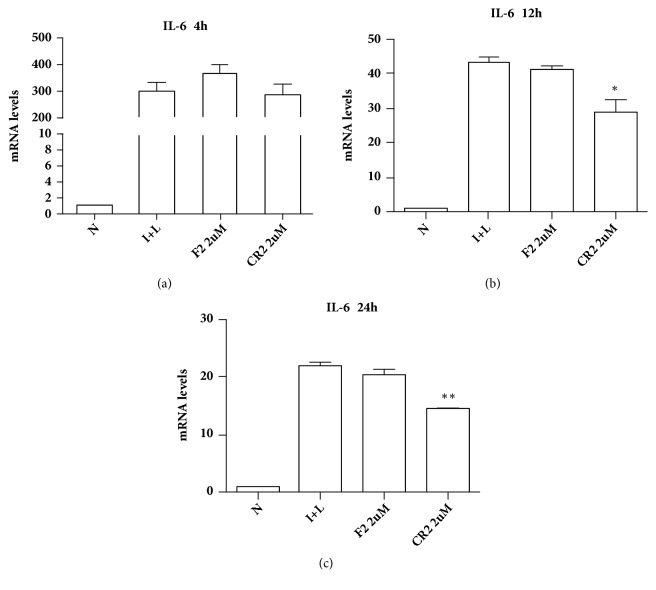
Effects of F2 and CR2 (2 *μ*M) on IL-6 expression in THP-1 cells. (a) IL-6 mRNA expression did not change after the addition of F2 and CR2 for 4 h. (b) IL-6 mRNA expression significantly decreased after the addition of CR2 for 12 h (*∗*P < 0.05), but there was no change after the addition of F2 for 12 h. (c) IL-6 mRNA expression significantly decreased after the addition of CR2 for 24 h (*∗∗*P < 0.01), but there was no change after the addition of F2 for 24 h (N: negative control; I+L: LPS and r-IFN).

There were no changes in IL-1*β*, TNF*α* and IL-8 mRNA expression after addition of F2 and CR2 at different concentrations (1 *μ*M, 10 *μ*M, and 100 *μ*M). IL-6 mRNA expression significantly decreased after the addition of CR2 (*∗*P < 0.05, *∗∗*P < 0.01), whereas there was no change after the addition of F2 (Figure 9). We then measured IL-6 mRNA expression after the addition of F2 and CR2 (2 *μ*M) for different times (4 h, 12 h, and 24 h). IL-6 mRNA expression significantly decreased after the addition of CR2 for 12 h and 24 h (*∗*P < 0.05), while there was no change after the addition of F2 at different times (Figure 10).

## 4. Discussion

MDR-TB has become a serious problem in TB treatment and control worldwide because of delayed diagnosis and its resistance to many first-line drugs. Previous researchers have focused on a better understanding of drug resistance mechanisms, facilitating the development of new tools for the rapid diagnosis of drug-resistant TB. There is an urgent need for the development of new biomarkers to improve the diagnosis and treatment of MDR-TB. [19]. Genomic and proteomic studies have identified many differentially expressed genes or proteins that may be useful for rapid diagnosis and potential novel targets for the treatment of MDR-TB [10, 20, 21]. Some of them are related to the virulence and pathogenicity of* M. tuberculosis*, and others are related to the immune status of the host.

Macrophages play a critical role in the host immune response, both innate and acquired, which serve as the major cell niche for the survival and killing of* M. tuberculosis*. Many studies have shown that the development of TB is associated with insufficient immune regulation of the host [22–24]. Furthermore, most lung damage is caused by host immunopathology, more than by tuberculous virulence factors. Therefore, in our study, we investigated the pathogenesis of MDR-TB from the host immune system and found novel biomarkers for the diagnosis and treatment of MDR-TB.

Peptidome analysis is a newly emerging discipline in proteomics that, in some cases, outperforms proteomics in its structural simplicity, operation convenience, study universality and property stability [17]. In a sense, the peptidome is inherited and developed from proteomics [25, 26]. Peptide analysis yields a more comprehensive picture of the nature and molecular functions of proteins by providing information regarding the synthesis, modification and degradation of proteins. In our study, we identified the differentially expressed peptides in the sera of patients with MDR-TB and DS-TB. Most peptides were involved in binding, catalytic activity and immune system processes.

Precursor proteins were predicted and analysed in the UniProt database. Three peptides that originated from the Fibrinogen *β* chain (FGB) were all significantly increased in MDR-TB. In our study, integrative analysis highlighted the complement and coagulation systems. LPS regulates many precursor proteins, four of which are correlated with organism survival. Wang et al. [10] reported increased complement C3, C4, and fibrinogen in MDR-TB patients compared with healthy controls. Moreover, the fibrinogen level in MDR-TB patients was higher than that of DS-TB patients. These results are similar to those of our study, indicating the presence of complement and coagulation cascade disorders in MDR-TB patients.

These important precursor proteins of four differentially expressed peptides include F2, CR2, COL5A2 and ITIH4. F2 is one component of the coagulation system that cleaves bonds after Arg and Lys, converts fibrinogen to fibrin and activates factors V, VII, VIII, XIII, and, in complex with thrombomodulin, protein C. CR2 is one component of the complement system. Studies indicate that human monocyte CR1 and CR3 mediate the phagocytosis of* M. tuberculosis*, and complement component C3 in serum acts as the major bacterium-bound ligand [27]. COL5A2 (Type V collagen) is a group I collagen (fibrillar forming collagen); it is a minor connective tissue component of nearly ubiquitous distribution. Type V collagen is a key determinant in the assembly of tissue-specific matrices. Sathyamoorthy et al. determined that* M. tuberculosis*-infected monocytes degraded collagen matrix in an MMP-dependent manner, and MMP neutralization decreased collagen degradation by 73% [28]. ITIH4 is a type II acute-phase protein that is involved in inflammatory responses to trauma and may also play a role in liver development or regeneration. However, these four peptides have rarely been studied in tuberculosis. We predict they may be used as diagnostic biomarkers and, furthermore, to establish a diagnostic model for MDR-TB.

Monocytic cells become adherent and acquire features of macrophages when infected with* M. tuberculosis *or exposed to the bacterial endotoxin LPS. Rivera-Marrero et al. determined that induced differentiation and/or activation of THP-1 cells by LPS, similar to infection with* M. tubercul*osis bacilli, resulted in the same regulation of specific genes [29]. Other studies have shown that LPS activates macrophages through TLR4 to enhance the production of reactive oxygen species (ROS), thus directly exerting bactericidal activities and facilitating the anti-infective immune response [30]. In our study, LPS and INF-r were used to stimulate THP-1 cells to produce* M. tuberculosis*-mediated macrophage activation. After the addition of CR2 peptide, IL-6 mRNA expression decreased significantly in dose-dependent manner and at different times. IL-6 is an important proinflammatory factor that promotes local inflammation. Studies have shown that IL-6 can inhibit the T cell response and TH1 differentiation [31]. IL-6 can also inhibit the autophagy of macrophages infected by TB, thereby promoting the escape of M*. tuberculosis* [32]. Therefore, CR2 peptide might regulate the proinflammatory cytokines secreted by macrophages to reduce local inflammatory response, promote enhancement of immune function, and facilitate the killing of* M. tuberculosis*. We propose the hypothesis that CR2 might have a therapeutic effect on MDR-TB. However, the F2 peptide has no effect on the expression of proinflammatory factors. Thus, we speculate that the CR2 peptide may be important in the pathogenesis of MDR-TB.

## 5. Conclusion

In conclusion, our study identified 40 differentially expressed peptides that may represent potential biomarkers for MDR-TB diagnosis, enabling a better understanding of the pathogenesis of MDR-TB. The functions of the differentially expressed peptides, especially CR2 in MDR-TB, require further investigation.

## Figures and Tables

**Table 1:** Characteristics of the 18 pulmonary tuberculosis patients.

Characteristics	MDR-TB	DS-TB
Cases	9	9
Sex		
Male	7	6
Female	2	3
Age, years (mean ± SD)	33.2 ± 9.38	30.2 ± 10.2
Prior history of TB treatment	0	0
Previous household exposure	3	5
Smoking	6	6
Alcoholism	2	3
Comorbidities	0	0

**Table 2:** List of peptides differentially expressed in multidrug-resistant tuberculosis compared with the control group (> 2-fold changes and P < 0.05).

Protein Name	Peptide	Mass	Fold Change	P
ATXN7	RRKRFDVL	1088.66	5.45	0.042
F2	TFGSGEADCGLRPLF	1625.75	5.18	0.036
PCLO	KPTILPKKK	1051.71	4.63	0.002
SYN2	VMDCS	553.19	4.02	0.027
DNAH2	KAEVEPLQR	1068.59	3.80	0.035
UNC13C	KSAVSGAIRLK	1128.70	3.60	0.048
CHD9	SSCSS	469.15	3.36	0.017
EPPK1	LVPAK	526.35	3.29	0.024
KIAA1211L	LARKK	614.42	3.26	0.004
TACC2	PPLPKAPSE	934.51	3.19	<0.001
COL5A2	GFPGNPGMKGEAGPTG	1472.67	3.01	0.039
UTP20	ILGKFVGKDQVTKLILPLKEILQNTTSLKLARKVHETLRRI	4709.85	3.00	0.010
TCOF1	KKSDKRKK	1016.65	2.95	0.036
OTOP2	FAGPVLGLLLFVVGLAV	1684.03	2.74	0.014
FCHO2	IKKRFAT	862.54	2.62	0.050
FGB	NDNEEGFFS	1079.38	2.50	0.025
TACC2	EANGV	488.22	2.49	0.033
POTEE	VGTSGDHDD	901.34	2.45	0.035
WDR81	VRLGLQAFL	1015.62	2.37	0.001
FGB	QGVNDNEEGFFS	1324.52	2.27	0.019
VPS13C	CQLFIQPA	918.46	2.22	0.015
FGB	KLKTM	619.37	2.09	0.039
GALNT10	SVATGDVIT	861.44	2.09	0.032
PLBD2	HLARALTRALALALVLALLVGPFLSGLAGAIPAPGG	3460.08	2.06	0.013
ASB14	IDEDFDTQ	981.39	-2.02	0.029
PRKDC	VKGAAGRTDLLRLRRR	1837.12	-2.06	0.018
CYP3A7-CYP3A51P	KVFPFL	749.45	-2.09	0.045
CR2	AGLLGVFLALVA	1142.71	-2.12	0.021
SVIL	HECDEGSEP	1001.34	-2.16	0.022
POTEE	VLDNKKRTALIKAVQ	1696.04	-2.19	0.013
SORCS3	RVIGGIAT	785.48	-2.27	0.006
ERN2	VSKALVHTGVAL	1193.71	-2.45	0.039
HMX3	AAPGAAGASVGAAAAT	1212.61	-2.55	0.042
TRPM1	GLKVIMGILLPPT	1350.83	-2.61	0.023
KYNU	GIRVAPVP	807.50	-2.81	0.035
ADAM15	NSCPCPGPAPAKT	1241.55	-3.33	0.026
OSBPL6	KRVTRRWR	1156.71	-3.36	0.039
ITIH4	QLGLPGPPDVPDHAAYHPF	2009.96	-3.82	0.025
C4A	TKDDPDAPLQPVTPLQLFEGR	2336.20	-4.54	0.005
LRP2	ICSCTAGFETNVFDRTSCL	2065.89	-4.73	0.040

## Data Availability

The data used to support the findings of this study are included within the article and the supplementary information file.

## Supplementary Materials

Supplementary Materials A total of 301 peptides were identified as differentially expressed peptides between the multiple drug-resistant tuberculosis and control sample groups. The details of the peptides are listed in Table S1.

## References

[1] Zumla A., George A., Sharma V., Herbert R. H. N., Oxley A., Oliver M. (2015). The WHO 2014 global tuberculosis report—further to go. *The Lancet Global Health*.

[2] Liang L., Wu Q., Gao L. (2012). Factors contributing to the high prevalence of multidrug-resistant tuberculosis: A study from China. *Thorax*.

[3] Kim S. J. (2005). Drug-susceptibility testing in tuberculosis: methods and reliability of results. *European Respiratory Journal*.

[4] Moore D. A. J., Mendoza D., Gilman R. H. (2004). Microscopic observation drug susceptibility assay, a rapid, reliable diagnostic test for multidrug-resistant tuberculosis suitable for use in resource-poor settings. *Journal of Clinical Microbiology*.

[5] Jain P., Hartman T. E., Eisenberg N. (2012). phi(2)GFP10, a high-intensity fluorophage, enables detection and rapid drug susceptibility testing of Mycobacterium tuberculosis directly from sputum samples. *Journal of Clinical Microbiology*.

[6] Ahmad S., Jaber A.-A., Mokaddas E. (2007). Frequency of embB codon 306 mutations in ethambutol-susceptible and -resistant clinical Mycobacterium tuberculosis isolates in Kuwait. *Tuberculosis*.

[7] Pulimood A. B., Peter S., Rook G. W. A., Donoghue H. D. (2008). In situ PCR for Mycobacterium tuberculosis in endoscopic mucosal biopsy specimens of intestinal tuberculosis and Crohn disease. *American Journal of Clinical Pathology*.

[8] Pule C. M., Sampson S. L., Warren R. M. (2016). Efflux pump inhibitors: Targeting mycobacterial efflux systems to enhance TB therapy. *Journal of Antimicrobial Chemotherapy*.

[9] Lata M., Sharma D., Deo N., Tiwari P. K., Bisht D., Venkatesan K. (2015). Proteomic analysis of ofloxacin-mono resistant Mycobacterium tuberculosis isolates. *Journal of Proteomics*.

[10] Wang C., Liu C.-M., Wei L.-L. (2016). A group of novel serum diagnostic biomarkers for multidrug-resistant tuberculosis by iTRAQ-2D LC-MS/MS and solexa sequencing. *International Journal of Biological Sciences*.

[11] Villanueva J., Shaffer D. R., Philip J. (2006). Differential exoprotease activities confer tumor-specific serum peptidome patterns. *The Journal of Clinical Investigation*.

[12] Bery A., Leung F., Smith C. R., Diamandis E. P., Kulasingam V. (2014). Deciphering the ovarian cancer ascites fluid peptidome. *Clinical Proteomics*.

[13] Dallas D. C., Guerrero A., Khaldi N. (2013). Extensive in vivo human milk peptidomics reveals specific proteolysis yielding protective antimicrobial peptides. *Journal of Proteome Research*.

[14] Nonaka A., Nakamura T., Hirota T. (2014). The milk-derived peptides Val-Pro-Pro and Ile-Pro-Pro attenuate arterial dysfunction in L-NAME-treated rats. *Hypertension Research*.

[15] Baum F., Fedorova M., Ebner J., Hoffmann R., Pischetsrieder M. (2013). Analysis of the endogenous peptide profile of milk: Identification of 248 mainly casein-derived peptides. *Journal of Proteome Research*.

[16] Boersema P. J., Raijmakers R., Lemeer S., Mohammed S., Heck A. J. R. (2009). Multiplex peptide stable isotope dimethyl labeling for quantitative proteomics. *Nature Protocols*.

[17] Qian Y., Zhang L., Rui C. (2017). Peptidome analysis of amniotic fluid from pregnancies with preeclampsia. *Molecular Medicine Reports*.

[18] Livak K. J., Schmittgen T. D. (2001). Analysis of relative gene expression data using real-time quantitative PCR and the 2(-Delta Delta C(T)) method. *Methods*.

[19] Islam M. M., Hameed H. M. A., Mugweru J. (2017). Drug resistance mechanisms and novel drug targets for tuberculosis therapy. *Journal of Genetics*.

[20] Singh A., Gopinath K., Sharma P. (2015). Comparative proteomic analysis of sequential isolates of Mycobacterium tuberculosis from a patient with pulmonary tuberculosis turning from drug sensitive to multidrug resistant. *Indian Journal of Medical Research, Supplement*.

[21] Yari S., Hadizadeh Tasbiti A., Ghanei M., Siadat S. D., Yari F., Bahrmand A. (2016). Proteome-scale MDR-TB-antibody responses for identification of putative biomarkers for the diagnosis of drug-resistant Mycobacterium tuberculosis. *International Journal of Mycobacteriology*.

[22] Rook G. A. W., Dheda K., Zumla A. (2005). Immune responses to tuberculosis in developing countries: implications for new vaccines. *Nature Reviews Immunology*.

[23] Rook G. A. W., Dheda K., Zumla A. (2006). Immune systems in developed and developing countries; implications for the design of vaccines that will work where BCG does not. *Tuberculosis*.

[24] Rook G. A. W., Lowrie D. B., Hernández-Pando R. (2007). Immunotherapeutics for tuberculosis in experimental animals: Is there a common pathway activated by effective protocols?. *The Journal of Infectious Diseases*.

[25] Ivanov V. T., Yatskin O. N. (2005). Peptidomics: A logical sequel to proteomics. *Expert Review of Proteomics*.

[26] Dallas D. C., Guerrero A., Parker E. A. (2015). Current peptidomics: Applications, purification, identification, quantification, and functional analysis. *Proteomics*.

[27] Schlesinger L. S., Bellinger-Kawahara C. G., Payne N. R., Horwitz M. A. (1990). Phagocytosis of *Mycobacterium tuberculosis* is mediated by human monocyte complement receptors and complement component C3. *The Journal of Immunology*.

[28] Sathyamoorthy T., Tezera L. B., Walker N. F. (2015). Membrane type 1 matrix metalloproteinase regulates monocyte migration and collagen destruction in tuberculosis. *The Journal of Immunology*.

[29] Rivera-Marrero C. A., Stewart J., Shafer W. M., Roman J. (2004). The down-regulation of cathepsin G in THP-1 monocytes after infection with Mycobacterium tuberculosis is associated with increased intracellular survival of bacilli. *Infection and Immunity*.

[30] Lv J., He X., Wang H. (2017). TLR4-NOX2 axis regulates the phagocytosis and killing of Mycobacterium tuberculosis by macrophages. *BMC Pulmonary Medicine*.

[31] Diehl S., Anguita J., Hoffmeyer A. (2000). Inhibition of Th1 differentiation by IL-6 is mediated by SOCS1. *Immunity*.

[32] Dutta R. K., Kathania M., Raje M., Majumdar S. (2012). IL-6 inhibits IFN-gamma induced autophagy in *Mycobacterium tuberculosis* H37Rv infected macrophages. *The International Journal of Biochemistry & Cell Biology*.

