# Supplementary material for: Significance of the Differential Peptidome in Multidrug-Resistant Tuberculosis
Source: Biomed Res Int. 2019 Jan 17;2019:5653424. doi: 10.1155/2019/5653424 (PMC6354167; doi:10.1155/2019/5653424)
Supplement: Supplementary Materials — A total of 301 peptides were identified as differentially expressed peptides between the multiple drug-resistant tuberculosis and control sample groups. The details of the peptides are listed in Table S1. [file 5653424.f1.docx]

**Table S1.** 301 peptides were identified as differentially expressed peptides between the multiple drug-resistant tuberculosis and control sample groups

| Protein Accession | Peptide | Mass | Length | DS-TB | MDR-TB | DS/MDR | P |
| --- | --- | --- | --- | --- | --- | --- | --- |
| Q86XH1 | KKAKKQKKGTKEKNK | 1771.1155 | 15 | 0 | 6.63108E-05 | ND | 0.138800504 |
| Q9H9J4-2 | HPKKQK | 764.4657 | 6 | 0 | 0.000114188 | ND | 0.136603884 |
| O94762 | PALLAKGITIVVS | 1280.8068 | 13 | 0 | 7.03715E-05 | ND | 0.125321347 |
| Q9UPN3 | ALAGLLVTY | 919.5378 | 9 | 3.26817E-05 | 0 | ND | 0.124674955 |
| P40879 | LDVSSVRGLKSILQEFIRIKVDVYI | 2889.6692 | 25 | 1.63409E-05 | 0 | ND | 0.124674955 |
| Q9NR82-2 | GFLVLI | 660.421 | 6 | 0 | 8.66142E-05 | ND | 0.117777191 |
| G3XAL9 | AVAVA | 429.2587 | 5 | 6.97318E-05 | 0 | ND | 0.107390393 |
| A4UGR9-2 | PPRRP | 621.371 | 5 | 0 | 3.46765E-05 | ND | 0.099962087 |
| Q09666 | TGPKIK | 642.4064 | 6 | 0 | 6.9353E-05 | ND | 0.099962087 |
| Q9UKN7 | LPAETK | 657.3697 | 6 | 0 | 5.09192E-05 | ND | 0.080608937 |
| P58107 | KVGLVG | 571.3693 | 6 | 0 | 7.74744E-05 | ND | 0.069132321 |
| A0A087WVH3 | LLHIGVPFVIIVANSPF | 1835.0709 | 17 | 3.72029E-05 | 0 | ND | 0.063465445 |
| P0DJD3-2 | KAVFGK | 648.3959 | 6 | 0 | 2.13989E-05 | ND | 0.061143561 |
| C9JIG9 | LLSGGSVLDIIKHIVAKGEHKSGVL | 2569.4956 | 25 | 0 | 4.27979E-05 | ND | 0.061143561 |
| Q14690 | FKVLK | 633.4213 | 5 | 0 | 4.17794E-05 | ND | 0.051069298 |
| Q9NRC6 | TSVAKK | 632.3857 | 6 | 4.17241E-05 | 0 | ND | 0.038884269 |
| A0A087WUR9 | HVIVK | 594.3853 | 5 | 4.5212E-06 | 0.000112151 | 24.80556845 | 0.097677985 |
| Q2LD37-4 | PGVLK | 512.3322 | 5 | 4.5212E-06 | 7.74744E-05 | 17.13580669 | 0.102204452 |
| H0YL34 | GEGDQAHREQGKEQAMFD | 2031.8704 | 18 | 2.2606E-06 | 2.90111E-05 | 12.83334929 | 0.053839225 |
| Q9Y520-2 | APVVK | 512.3322 | 5 | 4.5212E-06 | 5.80221E-05 | 12.83334929 | 0.053839225 |
| F8VWT9 | KPPAK | 539.3431 | 5 | 4.5212E-06 | 4.27979E-05 | 9.466044938 | 0.132457201 |
| Q3T8J9-3 | KKKGTK | 688.4595 | 6 | 4.5212E-06 | 4.17794E-05 | 9.240782925 | 0.072712443 |
| P63261 | EEEIAALVIDNG | 1313.635 | 12 | 1.37164E-05 | 8.55957E-05 | 6.240371433 | 0.104953523 |
| Q9Y4A5 | LRIAA | 542.354 | 5 | 1.82376E-05 | 0.000108941 | 5.973431645 | 0.078121939 |
| Q00975-2 | VWAAIKPGSSFGISVLRALRLLRIFKVT | 3097.8643 | 28 | 1.89653E-05 | 0.000113169 | 5.967182093 | 0.067570417 |
| Q9Y5H5 | LVLTLLLYTVLRC | 1518.9208 | 13 | 5.61682E-05 | 0.00032614 | 5.806489303 | 0.052368861 |
| B4E1Q4 | IKVFKT | 734.4691 | 6 | 9.0424E-06 | 4.99008E-05 | 5.518533053 | 0.087889715 |
| O15265 | RRKRFDVL | 1088.6566 | 8 | 4.6973E-05 | 0.000255769 | 5.445018499 | 0.042246604 |
| P00734 | TFGSGEADCGLRPLF | 1625.7507 | 15 | 2.27588E-05 | 0.000117901 | 5.18043274 | 0.036245477 |
| Q9NT68 | VCQTGWRGPGCNVAMETSCAD | 2183.8857 | 21 | 0.000101105 | 0.000492394 | 4.87013635 | 0.127878033 |
| Q9Y4C1 | KRAVK | 600.4071 | 5 | 1.89653E-05 | 8.98237E-05 | 4.736217459 | 0.126948749 |
| P78347-2 | KKHVGG | 624.3707 | 6 | 9.19525E-06 | 4.27979E-05 | 4.654345011 | 0.11311417 |
| Q9Y6V0-2 | KPTILPKKK | 1051.7117 | 9 | 9.14939E-06 | 4.23236E-05 | 4.625830956 | 0.002019306 |
| Q9Y6V0-2 | PVKKETK | 828.5068 | 7 | 5.68959E-06 | 2.59843E-05 | 4.566984196 | 0.14708105 |
| Q15334 | AQMVVAGTA | 846.4269 | 9 | 2.29117E-05 | 0.000101838 | 4.444822708 | 0.093644308 |
| Q8NHY0-3 | DSVVQGRGQKQLIIS | 1626.9053 | 15 | 3.26817E-05 | 0.000144636 | 4.425599865 | 0.080771864 |
| Q92793-2 | PHHVSPQT | 901.4406 | 8 | 0.000150286 | 0.000662945 | 4.411236146 | 0.140464456 |
| P02675 | QDGSVDFGRKWDP | 1490.679 | 13 | 0.000121379 | 0.000509835 | 4.200362954 | 0.069960105 |
| Q08722-2 | HGPLLISGLSILALA | 1473.8918 | 15 | 3.71265E-05 | 0.000152248 | 4.10080083 | 0.12958793 |
| Q92777-2 | VMDCS | 553.1876 | 5 | 0.000109999 | 0.000441816 | 4.016532379 | 0.026725552 |
| Q92736 | HMDDG | 573.1853 | 5 | 3.26817E-05 | 0.000129412 | 3.959766322 | 0.107602972 |
| P02671-2 | EEDFLAEGGGV | 1121.4877 | 11 | 6.01146E-05 | 0.000232772 | 3.872139346 | 0.139205107 |
| Q9P225-2 | KAEVEPLQR | 1068.5928 | 9 | 2.27588E-05 | 8.64337E-05 | 3.797807405 | 0.035430659 |
| Q5THK1-2 | AFAKK | 563.3431 | 5 | 1.37164E-05 | 5.09192E-05 | 3.712274299 | 0.133986436 |
| Q8N196 | TQVSNWFKNRR | 1434.748 | 11 | 0.000572869 | 0.002092055 | 3.651891913 | 0.139751744 |
| E9PB90 | TEKGDFLALNLGGT | 1434.7355 | 14 | 0.001087468 | 0.00395946 | 3.640990333 | 0.136674547 |
| Q7Z5P9 | TATTILSTGA | 934.4971 | 10 | 6.85822E-06 | 2.49504E-05 | 3.63802372 | 0.085492061 |
| O15020 | STMDANG | 694.2592 | 7 | 1.37164E-05 | 4.99008E-05 | 3.63802372 | 0.085492061 |
| Q8NB66 | KSAVSGAIRLK | 1128.6979 | 11 | 0.000107088 | 0.000385992 | 3.604454985 | 0.048468913 |
| P54802 | VAAAVGVLLLAGAGG | 1237.7394 | 15 | 3.26817E-05 | 0.000113169 | 3.462769987 | 0.094242645 |
| Q8IZD9 | GSNSTLSGSASSGV | 1209.5474 | 14 | 3.74903E-05 | 0.000129566 | 3.455990784 | 0.088462307 |
| A0AVI2-2 | DIFSPDDFLGVLELD | 1693.8086 | 15 | 0.022716259 | 0.077777181 | 3.423855138 | 0.100491957 |
| Q3L8U1-2 | SSCSS | 469.1479 | 5 | 0.000124023 | 0.000416287 | 3.356533708 | 0.016605047 |
| Q9H3S1 | HFVTVTVLFALVLSGALIILVA | 2295.397 | 22 | 6.4483E-05 | 0.000213989 | 3.318539659 | 0.143114559 |
| O95425-4 | KALAK | 529.3588 | 5 | 2.34865E-05 | 7.74744E-05 | 3.298678542 | 0.053705097 |
| P58107 | LVPAK | 526.3478 | 5 | 3.25289E-05 | 0.000106904 | 3.286445955 | 0.023750532 |
| O75151 | ARKNGGGSGKSAGKRLLKRAAKNSVD | 2625.4897 | 26 | 1.89653E-05 | 6.22501E-05 | 3.282318609 | 0.063342074 |
| Q6NV74 | LARKK | 614.4227 | 5 | 2.34865E-05 | 7.64559E-05 | 3.255315134 | 0.004231123 |
| Q75N90 | TCYGAIEKGSCAR | 1357.6118 | 13 | 6.22224E-05 | 0.000201982 | 3.246128642 | 0.111999868 |
| Q70CQ1-2 | KHVGRL | 708.4395 | 6 | 1.89653E-05 | 6.12317E-05 | 3.228617656 | 0.05394895 |
| Q4ZG55 | APAAGT | 486.2438 | 6 | 5.1647E-05 | 0.00016628 | 3.219539751 | 0.071205339 |
| P23634-3 | IIGITVLVVAVPEGLPLAVTISLAYSVKK | 2962.8086 | 29 | 3.79306E-05 | 0.000121291 | 3.197701978 | 0.120348413 |
| O95359-3 | PPLPKAPSE | 934.5123 | 9 | 0.000137434 | 0.000438447 | 3.190243964 | 0.000247239 |
| Q9BU23-2 | GALVALGALLLSPLRHPVIYLLLWAAYL | 3015.804 | 28 | 3.26817E-05 | 0.00010403 | 3.183109029 | 0.09320961 |
| P20264 | GGGGGGGGGGG | 645.2466 | 11 | 0.000102414 | 0.000324453 | 3.168063277 | 0.072921599 |
| Q6MZP7-2 | LILTT | 559.3581 | 5 | 4.6973E-05 | 0.000147846 | 3.147466115 | 0.092139341 |
| Q6ZTR5 | KKVVM | 603.3778 | 5 | 3.72029E-05 | 0.000117063 | 3.146597925 | 0.13159384 |
| P05997 | GFPGNPGMKGEAGPTG | 1472.6718 | 16 | 0.000652167 | 0.001963794 | 3.011182661 | 0.038851306 |
| O75691 | ILGKFVGKDQVTKLILPLKEILQNTTSLKLARKVHETLRRI | 4709.8525 | 41 | 2.81605E-05 | 8.45773E-05 | 3.00339672 | 0.009650134 |
| O95248 | AGGSAGGP | 572.2554 | 8 | 0.000358959 | 0.00107788 | 3.00279356 | 0.074222687 |
| A0A0J9YWL0 | KLGLALD | 728.4432 | 7 | 2.34865E-05 | 7.03715E-05 | 2.996253689 | 0.143077478 |
| E5RI98 | KLAADEDDDDD | 1220.468 | 11 | 1.63409E-05 | 4.84633E-05 | 2.965773653 | 0.143532434 |
| X6REW1 | PPAVTPE | 709.3646 | 7 | 3.69155E-05 | 0.000109451 | 2.964891084 | 0.091501548 |
| Q13428-7 | KKSDKRKK | 1016.6454 | 8 | 2.34865E-05 | 6.9353E-05 | 2.952890281 | 0.036376859 |
| Q14624 | GVLSSRKLGLPGPPDVPDHA | 2011.085 | 20 | 3.72029E-05 | 0.000108941 | 2.928299241 | 0.098513952 |
| Q10571 | KAPPP | 508.3009 | 5 | 9.92725E-05 | 0.000286894 | 2.889963517 | 0.143845419 |
| Q8TEP8 | KTATE | 548.2806 | 5 | 0.000183965 | 0.000529231 | 2.876800479 | 0.136591137 |
| Q92793-2 | QPRKKIFK | 1043.6603 | 8 | 0.000119807 | 0.000343401 | 2.866280127 | 0.143817668 |
| Q9UQC9 | LSRKKRADK | 1100.6777 | 9 | 1.89653E-05 | 5.41288E-05 | 2.854096747 | 0.104515813 |
| P50570-2 | KTIMH | 628.3367 | 5 | 2.27588E-05 | 6.41066E-05 | 2.81677568 | 0.062590951 |
| H0Y9V3 | SLPTKKITKK | 1142.7386 | 10 | 6.52106E-05 | 0.000181504 | 2.783348844 | 0.085192899 |
| A0A087WXD3 | IIYIII | 746.4942 | 6 | 2.80077E-05 | 7.74744E-05 | 2.766182616 | 0.104816115 |
| Q14624 | QLGLPGPPDVPDHA | 1411.7095 | 14 | 8.72065E-05 | 0.000241026 | 2.763849183 | 0.132271953 |
| Q7RTS6 | FAGPVLGLLLFVVGLAV | 1684.0327 | 17 | 5.61682E-05 | 0.00015393 | 2.740522651 | 0.014124531 |
| P20929-3 | EKLYQEAWN | 1179.556 | 9 | 0.000102157 | 0.000277741 | 2.718752306 | 0.125995141 |
| Q02388-2 | TAYRLAW | 879.4603 | 7 | 0.000564211 | 0.00153269 | 2.716518237 | 0.106476871 |
| G3V198 | LAFKCI | 693.3884 | 6 | 0.000255775 | 0.000693578 | 2.711674019 | 0.11390876 |
| A0A096LNW2 | CPQDGCP | 718.2415 | 7 | 5.61682E-05 | 0.000151739 | 2.701512984 | 0.116820737 |
| Q8TBG4-2 | KRVGN | 572.3394 | 5 | 5.10722E-05 | 0.000137533 | 2.692918998 | 0.095100049 |
| E9PHY5 | GAQKIPG | 669.3809 | 7 | 8.44816E-06 | 2.27357E-05 | 2.691202387 | 0.068686378 |
| Q9P2D7 | PPGVKLVIEAVCIMKGIKPKKV | 2346.4258 | 22 | 2.34865E-05 | 6.22501E-05 | 2.650465428 | 0.134125447 |
| Q0JRZ9 | IKKRFAT | 862.5388 | 7 | 3.26817E-05 | 8.55957E-05 | 2.619069021 | 0.049849354 |
| P00451 | GRKYKK | 778.4813 | 6 | 2.34865E-05 | 6.12317E-05 | 2.607102021 | 0.076901012 |
| Q99570 | KAAMLF | 679.3727 | 6 | 0.000408243 | 0.001056177 | 2.587127884 | 0.092481875 |
| P68366-2 | SYEDEDEGE | 1128.373 | 9 | 0.000213114 | 0.000545414 | 2.559256247 | 0.127784421 |
| A0A087WXD3 | QTFTTQ | 724.3392 | 6 | 5.07665E-05 | 0.000127195 | 2.505482647 | 0.086838241 |
| J3QKL0 | GAGGCPA | 531.2111 | 7 | 0.003576961 | 0.008952789 | 2.502903494 | 0.149982936 |
| P02675 | NDNEEGFFS | 1079.3807 | 9 | 0.00015021 | 0.000375783 | 2.501717613 | 0.025181831 |
| P02671-2 | DSEEGDFLAEGGGVR | 1536.6692 | 15 | 3.21651E-05 | 8.03493E-05 | 2.498029101 | 0.113051413 |
| O95359-3 | EANGV | 488.2231 | 5 | 0.00014608 | 0.000363486 | 2.488258041 | 0.032514024 |
| Q8IVL0-2 | KKTSKIA | 774.4963 | 7 | 2.80077E-05 | 6.9353E-05 | 2.476213933 | 0.089975638 |
| A0A087WUP5 | VLFPNLKT | 930.5538 | 8 | 3.46549E-05 | 8.55957E-05 | 2.469942936 | 0.120963905 |
| Q68CP9-3 | GPITKHIRLTAALILKNIGK | 2156.3521 | 20 | 2.81605E-05 | 6.9353E-05 | 2.462773215 | 0.066487392 |
| Q6S8J3 | VGTSGDHDD | 901.3413 | 9 | 9.26435E-05 | 0.000227177 | 2.452159266 | 0.034567669 |
| Q8IYE0 | LVKLLELA | 897.5898 | 8 | 3.26817E-05 | 7.84928E-05 | 2.401733644 | 0.140371553 |
| P19338 | KKAAATSAKK | 1002.6185 | 10 | 6.98847E-05 | 0.00016628 | 2.379343731 | 0.144716379 |
| A0A0J9YXM6 | VRLGLQAFL | 1015.6178 | 9 | 0.000139311 | 0.000330718 | 2.373955096 | 0.00097391 |
| M0QZD8 | SGLGLQGLAVL | 1026.6073 | 11 | 7.35254E-05 | 0.000174221 | 2.369529419 | 0.126699087 |
| Q92887 | KSRTKRS | 861.5144 | 7 | 3.79306E-05 | 8.98237E-05 | 2.368108729 | 0.080328335 |
| O43313 | SSDTETQT | 867.3458 | 8 | 0.001529363 | 0.003593472 | 2.349652217 | 0.076021959 |
| Q9H329-2 | KKQMKIGP | 928.5528 | 8 | 3.26817E-05 | 7.64559E-05 | 2.339408063 | 0.063999902 |
| Q9Y4F1 | ITVWLDLLKPIVKQIRRPKHVVVKFVVK | 3351.1162 | 28 | 4.24518E-05 | 9.69266E-05 | 2.283217335 | 0.053703223 |
| Q9ULI4 | AGEAAGGPLCLS | 1044.491 | 12 | 0.000188506 | 0.000429475 | 2.278309533 | 0.148871138 |
| P02675 | QGVNDNEEGFFS | 1324.5208 | 12 | 3.72029E-05 | 8.45773E-05 | 2.273403188 | 0.019478632 |
| Q8TEP8 | KTFLAWGGVR | 1133.6345 | 10 | 0.003737837 | 0.008485822 | 2.270249436 | 0.062840892 |
| O75151 | KTVKMPKPSKIPKPPKPPKPPR | 2475.5239 | 22 | 7.44059E-05 | 0.000166622 | 2.239360246 | 0.067813432 |
| A0A088AWL3 | TKGKPYDGITTIK | 1420.7925 | 13 | 6.98847E-05 | 0.000155967 | 2.23177982 | 0.111564513 |
| Q709C8-2 | CQLFIQPA | 918.4633 | 8 | 0.000222988 | 0.000495148 | 2.220510774 | 0.014700045 |
| P98088 | TSPSISTSEPVT | 1204.5823 | 12 | 0.001061063 | 0.002350366 | 2.215106227 | 0.13405952 |
| Q7Z5P9 | VTGGPSTAAS | 846.4083 | 10 | 0.000116283 | 0.000256941 | 2.209623834 | 0.113608501 |
| H0Y4R2 | QTGTAEEFANRL | 1335.6418 | 12 | 0.000555712 | 0.001221717 | 2.198469589 | 0.141627039 |
| Q16851 | FLPVKT | 703.4268 | 6 | 5.1647E-05 | 0.000113169 | 2.191207218 | 0.082176879 |
| Q96N64 | HAKAREVLKIAKEKAQK | 1947.1741 | 17 | 2.80077E-05 | 6.12317E-05 | 2.186245249 | 0.133619171 |
| E7ENN3 | EFDAG | 537.207 | 5 | 4.17241E-05 | 9.06617E-05 | 2.172884328 | 0.073052801 |
| O95278-4 | RPAGTAAGDGAL | 1055.5359 | 12 | 9.23744E-05 | 0.000200467 | 2.170160055 | 0.05492277 |
| G3V1B5 | SIITGF | 636.3483 | 6 | 3.26817E-05 | 7.03715E-05 | 2.153235477 | 0.13601823 |
| Q96PY6 | PASGQNSISVMPAQKITKP | 1953.0353 | 19 | 0.000110612 | 0.000237154 | 2.144019409 | 0.134576939 |
| P02462 | KGDRGP | 628.3292 | 6 | 5.09194E-05 | 0.000108941 | 2.139486412 | 0.097560642 |
| A0A087WU49 | PPRKPASNLVGVLLGLLVPVVVVLLAVTRECIYRT | 3754.2422 | 35 | 3.79306E-05 | 8.06839E-05 | 2.127147322 | 0.056879537 |
| Q7RTP6 | RGFLAAM | 764.4003 | 7 | 3.26817E-05 | 6.9353E-05 | 2.122072686 | 0.089763175 |
| P13569-2 | IFDFIQLLLIVIGAIAVVAVLQPYIFVATVPVIVAFIMLRAYFLQT | 5120.9927 | 46 | 1.86779E-05 | 3.92464E-05 | 2.101222616 | 0.06604893 |
| P02675 | KLKTM | 619.3727 | 5 | 2.12259E-05 | 4.44026E-05 | 2.091909639 | 0.038577972 |
| Q86SR1 | SVATGDVIT | 861.4443 | 9 | 0.00011421 | 0.000238623 | 2.089324281 | 0.031847652 |
| Q00975-2 | GDKVMSECSLEKN | 1438.6432 | 13 | 0.000182783 | 0.000381847 | 2.08907228 | 0.071200586 |
| P01024 | RIHWESASL | 1079.5511 | 9 | 6.05366E-05 | 0.000126357 | 2.087278496 | 0.061385644 |
| O43432 | EVLPL | 569.3424 | 5 | 3.72029E-05 | 7.74744E-05 | 2.082480143 | 0.091051453 |
| Q6MZP7-2 | IILNKVSQT | 1014.6073 | 9 | 4.66244E-05 | 9.67913E-05 | 2.075978288 | 0.130472501 |
| P14866 | MAAAGGGGGGGR | 917.4137 | 12 | 3.73558E-05 | 7.74744E-05 | 2.073958992 | 0.080869338 |
| P41594-2 | TGVSGDTIL | 861.4443 | 9 | 3.70501E-05 | 7.64559E-05 | 2.063583022 | 0.122226362 |
| Q8NHP8 | HLARALTRALALALVLALLVGPFLSGLAGAIPAPGG | 3460.0808 | 36 | 4.71258E-05 | 9.69266E-05 | 2.056762431 | 0.013290328 |
| C9JAQ0 | CHGAGT | 544.2064 | 6 | 0.000118391 | 0.000237664 | 2.007453806 | 0.064588334 |
| Q15149-8 | TGSVAGVYLPGSRQT | 1491.7681 | 15 | 0.000528413 | 0.001058744 | 2.003630746 | 0.092638268 |
| P68363 | TVVPGGDLAKV | 1054.6022 | 11 | 0.000181072 | 0.000362688 | 2.003010766 | 0.132947296 |
| Q9H6K5-2 | QSMPPTQ | 819.3433 | 7 | 6.90042E-05 | 0.000137353 | 1.990501577 | 0.026921108 |
| P02671-2 | GEGDFLAEGGGV | 1106.4879 | 12 | 5.646E-05 | 0.000111423 | 1.973481328 | 0.036134082 |
| P02671-2 | TVTKT | 576.3483 | 5 | 0.000289478 | 0.00056939 | 1.966956435 | 0.060707928 |
| Q00975-2 | QCDAE | 564.1849 | 5 | 0.000139793 | 0.000273499 | 1.95646271 | 0.066955232 |
| Q96JG9 | NPSGLEGGTVEGGKVACGPAQGSPG | 2225.0381 | 25 | 5.10722E-05 | 9.98015E-05 | 1.954124764 | 0.106897647 |
| O95810 | GAVEGKEELPDENKSLEETLH | 2323.1179 | 21 | 2.54597E-05 | 4.93915E-05 | 1.93998949 | 0.08649347 |
| Q96T76-9 | VLQGLKALSLCVALPPGLAVSVLKA | 2459.4912 | 25 | 4.24518E-05 | 8.17024E-05 | 1.924592792 | 0.135868577 |
| Q96PN7-2 | QKAAFAAEMAAT | 1208.5859 | 12 | 0.000108454 | 0.000208568 | 1.923104672 | 0.091979259 |
| P01024 | HWESASL | 810.366 | 7 | 4.18088E-05 | 7.96672E-05 | 1.905513697 | 0.013926175 |
| Q9BZ95-5 | EDAFDNNSDIAEDGGQTP | 1893.75 | 18 | 0.000102682 | 0.000194057 | 1.889890524 | 0.026952021 |
| H0YLX2 | KTGDGLEGAEP | 1072.5037 | 11 | 0.001030781 | 0.001942337 | 1.884334349 | 0.086684303 |
| H0YNG5 | GVRLLRSLVNLKQVIIHAAH | 2236.3643 | 20 | 7.98076E-05 | 0.000148864 | 1.865290603 | 0.110755859 |
| P0C0L4-2 | DDPDAPLQPVTPLQLFEGR | 2107.0586 | 19 | 3.72029E-05 | 6.9353E-05 | 1.864181458 | 0.147335815 |
| Q8WXG9 | YISSHSDFI | 1067.4923 | 9 | 0.000512944 | 0.000951942 | 1.855841428 | 0.144604794 |
| Q8IZQ1-2 | HPAKKF | 726.4177 | 6 | 4.63982E-05 | 8.55957E-05 | 1.844807647 | 0.093247051 |
| P02671-2 | KTFPGFFS | 943.4439 | 8 | 0.001062115 | 0.001947996 | 1.834072294 | 0.142048136 |
| Q15375 | GRLKLPGKRDVAV | 1407.8673 | 13 | 0.000102719 | 0.000187602 | 1.826351603 | 0.07713671 |
| A0A0A0MSU4 | VLSILGNPSVV | 1096.6492 | 11 | 5.55934E-05 | 0.00010082 | 1.813523075 | 0.02524147 |
| Q15942 | AAPRPSPAISVSVS | 1379.7408 | 14 | 8.50916E-05 | 0.000154107 | 1.811076739 | 0.118698306 |
| P49753 | HGRRKPQI | 990.5835 | 8 | 3.79306E-05 | 6.79156E-05 | 1.790523086 | 0.022014643 |
| F8W8Y7 | EVGEGAMGTA | 920.3909 | 10 | 0.000144443 | 0.00025475 | 1.763668503 | 0.003132773 |
| Q9Y6V0 | PGRGAE | 585.287 | 6 | 8.78349E-05 | 0.000154614 | 1.760280215 | 0.098310769 |
| P0C0L4-2 | VFVDHHLAP | 1047.5138 | 9 | 0.000477366 | 0.000833229 | 1.745469574 | 0.108903688 |
| O95359-3 | SSTLKRTKKPRPPSLKKKQTTKK | 2665.6443 | 23 | 5.1647E-05 | 8.98237E-05 | 1.739185049 | 0.138107521 |
| Q07092-2 | VIGPK | 512.3322 | 5 | 3.26817E-05 | 5.65847E-05 | 1.731384994 | 0.09854126 |
| Q8NFD5-3 | QQQQQQH | 923.421 | 7 | 0.000289526 | 0.000501096 | 1.730743466 | 0.031302881 |
| Q5VZU9 | KNWVQTLRPVSAKT | 1626.9205 | 14 | 8.74528E-05 | 0.000150631 | 1.722420749 | 0.113448554 |
| P78527-2 | ALVVEKMLKALKLNSNEARLKFPRLLQII | 3348.0205 | 29 | 5.1647E-05 | 8.88053E-05 | 1.719465537 | 0.093987651 |
| O00410-2 | EDGNNQWPE | 1087.4207 | 9 | 9.36361E-05 | 0.000158129 | 1.688763731 | 0.092921514 |
| Q9Y485 | SIHPMDGSLL | 1068.5273 | 10 | 6.70493E-05 | 0.000113002 | 1.685357385 | 0.033763887 |
| P07585-2 | DFCPPG | 634.2421 | 6 | 7.86397E-05 | 0.000130759 | 1.662764385 | 0.055704058 |
| H7C0L5 | NVHSAGAAGSRMNFRPGVLS | 2027.0118 | 20 | 0.000662062 | 0.001097147 | 1.657166557 | 0.120210465 |
| H0Y4I5 | LSVLAPEALAGLPALRRLSL | 2059.2517 | 20 | 4.75627E-05 | 7.84707E-05 | 1.649838385 | 0.005832719 |
| Q15154 | GAGAGT | 432.1968 | 6 | 0.000146629 | 0.000241886 | 1.649646898 | 0.077744187 |
| H0Y2L4 | VDLFKRRVVRRLASLKTRRCRL | 2740.6711 | 22 | 3.75668E-05 | 6.17409E-05 | 1.643498167 | 0.023963331 |
| P78509-3 | EPCPS | 531.1999 | 5 | 0.000385746 | 0.000628769 | 1.630009773 | 0.148199849 |
| O15230 | KVSVR | 587.3755 | 5 | 0.0008601 | 0.001400214 | 1.627967482 | 0.09816205 |
| Q9BTC0-1 | IAPKT | 528.3271 | 5 | 8.78349E-05 | 0.000142941 | 1.627385457 | 0.071816265 |
| Q400G9 | PPGGP | 423.2118 | 5 | 4.68279E-05 | 7.61687E-05 | 1.626564976 | 0.066615933 |
| E5RIJ0 | PHKIK | 621.3962 | 5 | 0.000191963 | 0.000309885 | 1.614295852 | 0.119483299 |
| Q8IZD9 | LHFDAFHHPLGD | 1404.6575 | 12 | 0.000125402 | 0.00020155 | 1.607232953 | 0.070319185 |
| Q9UPN3 | TALFA | 521.2849 | 5 | 0.00018544 | 0.000292721 | 1.57852047 | 0.024601026 |
| P78509-3 | DPGFSGPACEM | 1109.4158 | 11 | 0.000111609 | 0.000175671 | 1.573989748 | 0.119056796 |
| G3V2A4 | RAAPHR | 706.3987 | 6 | 0.000197929 | 0.000309995 | 1.566192017 | 0.127688491 |
| A0A087X011 | KKVIVGGVDLLAKAEEQEKLL | 2279.3464 | 21 | 0.000107815 | 0.00016628 | 1.542264693 | 0.014018731 |
| A0A0A0MS97 | EGVNKGIYFSYPCRRHSCAVVNIP | 2708.3315 | 24 | 0.000276049 | 0.000425449 | 1.541207027 | 0.074427109 |
| A0A0G2JR66 | ARGPSASTTK | 974.5145 | 10 | 0.00031429 | 0.000480394 | 1.528506671 | 0.121960934 |
| A0A0C4DGW7 | RTSSLRSEPPKT | 1357.7313 | 12 | 0.000172587 | 0.000262691 | 1.522080767 | 0.095313668 |
| Q86UR5-6 | EQKQWSSRSRSEPPR | 1856.9241 | 15 | 7.03722E-05 | 0.000106275 | 1.51018959 | 0.039528254 |
| P62328 | SDKPDMAEIEKFDKSKLKKTETQE | 2866.427 | 24 | 6.8295E-05 | 0.000102725 | 1.504141162 | 0.140699406 |
| Q9H4A3-7 | AHPCGG | 540.2114 | 6 | 0.000282892 | 0.000423738 | 1.497877227 | 0.034145147 |
| E9PNE6 | NTTIPTKQT | 1002.5345 | 9 | 9.19159E-05 | 0.000137353 | 1.494332985 | 0.025006371 |
| Q13129 | ELGGD | 489.2071 | 5 | 0.000184501 | 0.00027412 | 1.485732369 | 0.045163426 |
| Q9NWZ5-4 | RTLSVLKS | 902.5549 | 8 | 0.00056504 | 0.000829677 | 1.4683525 | 0.041961176 |
| Q9Y5A7 | EMTPYLDIANQTGRSIRIPPSERKALMLAMGYHEK | 4016.0435 | 35 | 0.000115017 | 0.000165146 | 1.435835295 | 0.047601651 |
| P02462 | DGIPGSAGEKGEPGLP | 1479.7205 | 16 | 0.000215489 | 0.0003011 | 1.397282682 | 0.035687318 |
| P01023 | VGFYESDVMG | 1102.4641 | 10 | 0.000149252 | 0.000208401 | 1.396302703 | 0.05259502 |
| P02671-2 | DSGEGDFLAEGGG | 1209.4785 | 13 | 9.0888E-05 | 0.000126499 | 1.391808989 | 0.078786321 |
| Q8TDZ2-4 | SCCPPQEASMASPTS | 1494.5789 | 15 | 0.000517115 | 0.000714601 | 1.381900157 | 0.085437945 |
| Q13009-2 | VFKTAVVLVYKDGSKQKKK | 2165.2935 | 19 | 5.1647E-05 | 6.9353E-05 | 1.342826986 | 0.149320091 |
| Q8IVF5-2 | KLTKGT | 646.4014 | 6 | 4.71258E-05 | 6.22501E-05 | 1.320934299 | 0.131417132 |
| F2Z357 | EEDYIPYPSVH | 1347.5983 | 11 | 0.001117857 | 0.000738075 | -1.514557834 | 0.110762056 |
| Q7Z3U7-2 | VAVGQPLAV | 852.5069 | 9 | 9.26435E-05 | 6.10643E-05 | -1.517146089 | 0.11905673 |
| A0A087WW67 | NPSHDTF | 816.3402 | 7 | 0.00040882 | 0.000268103 | -1.524859855 | 0.100319881 |
| Q9P227 | YKQILTKKGKKAG | 1461.9031 | 13 | 0.000138889 | 9.06617E-05 | -1.531945945 | 0.03903591 |
| A0A096LNW2 | SCNGGP | 533.1904 | 6 | 0.000120957 | 7.74744E-05 | -1.561250546 | 0.115628645 |
| Q6ZMW3 | RCLKTYAI | 966.532 | 8 | 0.000478183 | 0.000304651 | -1.569609004 | 0.038344672 |
| Q9UIS9 | KKRKKPSR | 1026.6774 | 8 | 0.000550783 | 0.00035019 | -1.572811132 | 0.083178255 |
| Q63HN8-4 | ALINIIKPPVRDPK | 1572.9714 | 14 | 7.68193E-05 | 4.84633E-05 | -1.585102435 | 0.100850227 |
| Q9Y251-2 | AYLRFGGTKT | 1112.5978 | 10 | 9.94253E-05 | 6.26691E-05 | -1.586512203 | 0.023879396 |
| Q01668-3 | TSQPNSSKQT | 1076.5098 | 10 | 0.000444474 | 0.000278934 | -1.593475853 | 0.072602876 |
| O15066-2 | ELKLKHLII | 1105.7223 | 9 | 0.160817299 | 0.10006282 | -1.607163364 | 0.026000374 |
| A0A087WXK5 | GLLGVNGAGKT | 985.5556 | 11 | 0.000185076 | 0.00011393 | -1.624468597 | 0.072488073 |
| P51532-4 | EKDKKGKGGTKT | 1275.7146 | 12 | 0.000184712 | 0.000112074 | -1.648130617 | 0.144090704 |
| G3V1B5 | KNSVGSGR | 803.4249 | 8 | 0.001099066 | 0.000662561 | -1.658815986 | 0.055344138 |
| Q8TF62 | QKCIWLVILLTTVASVMPV | 2113.2043 | 19 | 8.36011E-05 | 4.99008E-05 | -1.675347609 | 0.116652421 |
| B4DYV8 | EDMEVSHTQ | 1074.4288 | 9 | 0.009046864 | 0.005388147 | -1.679030752 | 0.104710522 |
| Q9UKN7 | PRVHT | 608.3394 | 5 | 0.001215353 | 0.000723822 | -1.679077464 | 0.13994241 |
| O94759-2 | LSWEIYLKENY | 1456.7238 | 11 | 0.000769977 | 0.00045426 | -1.695015246 | 0.040182252 |
| A0A087WTM7 | KVLADK | 672.417 | 6 | 9.3524E-05 | 5.51472E-05 | -1.695896957 | 0.041285445 |
| Q9NRC6 | AALLESRKNPE | 1226.6619 | 11 | 0.00019224 | 0.000113002 | -1.701208722 | 0.075255387 |
| Q9P218 | KATVGGL | 644.3857 | 7 | 0.000563653 | 0.000330203 | -1.706992626 | 0.138449524 |
| A0A087X1R1 | ELVTGATGTGDLTRKEPTE | 1973.9905 | 19 | 0.000179311 | 0.000104713 | -1.712394874 | 0.076216305 |
| Q6IN85-2 | EDEEEMWF | 1113.3961 | 8 | 0.000226073 | 0.000131701 | -1.716564273 | 0.141257503 |
| Q8IYW2 | EEFENCV | 868.3273 | 7 | 7.49726E-05 | 4.31383E-05 | -1.737957737 | 0.085148893 |
| A0A087WU49 | SPAGSPPIPLWKR | 1404.7877 | 13 | 7.87161E-05 | 4.52538E-05 | -1.739437779 | 0.12295987 |
| Q15772-1 | ATATNELGQATCAAS | 1407.63 | 15 | 0.00104009 | 0.000594066 | -1.750797961 | 0.010076178 |
| O75807 | DDSEAALGEAESD | 1307.5 | 13 | 0.000496645 | 0.000282995 | -1.754964251 | 0.132779151 |
| E5RJ97 | SEKAGFNYES | 1130.488 | 10 | 0.000198851 | 0.000112989 | -1.75991351 | 0.035641651 |
| Q9UPN3 | EKSRSGGRKSL | 1203.6683 | 11 | 0.000268001 | 0.000151893 | -1.764403445 | 0.127373018 |
| Q9UPA5 | KKTRVPTK | 956.613 | 8 | 0.000482512 | 0.000272011 | -1.773867821 | 0.021694781 |
| Q14573 | GLVDD | 517.2384 | 5 | 0.00030521 | 0.00017125 | -1.782251054 | 0.149111634 |
| G3V347 | SWGQNTQ | 819.3511 | 7 | 0.001134671 | 0.00063255 | -1.793806339 | 0.009940055 |
| Q6ZS81-5 | STEYSVSGGIGTGA | 1284.5834 | 14 | 0.000856543 | 0.000475188 | -1.802535647 | 0.090077764 |
| O60840-2 | TGSMTETQGDEDEEE | 1656.5945 | 15 | 0.000486618 | 0.000269486 | -1.805728863 | 0.069625617 |
| Q9NU22 | ARLNAALATPAKEMG | 1512.8082 | 15 | 0.000244615 | 0.000132545 | -1.845528557 | 0.053905689 |
| Q9GZR2 | KIARKQLGQ | 1040.6454 | 9 | 7.90035E-05 | 4.27979E-05 | -1.84596823 | 0.072154446 |
| Q8IU80-1 | WHLTVPSLDYGL | 1399.7136 | 12 | 0.000383556 | 0.000206706 | -1.855565568 | 0.055109714 |
| P15924-2 | ESKNQCTQ | 936.3971 | 8 | 0.0015337 | 0.000813075 | -1.886296304 | 0.073575921 |
| H0Y3P2 | KPAKT | 543.338 | 5 | 9.71647E-05 | 5.09192E-05 | -1.908213123 | 0.135700149 |
| P02671-2 | TADSGEGDFLAEGGGV | 1537.6532 | 16 | 0.000621067 | 0.000323641 | -1.919001441 | 0.09653657 |
| A0A087WXB1 | RVRTRGGVGKA | 1155.6948 | 11 | 0.000135248 | 6.98623E-05 | -1.935926037 | 0.105262643 |
| Q6ZNJ1-3 | YESFEDPAGT | 1114.4454 | 10 | 0.00027617 | 0.000139544 | -1.979085507 | 0.083280522 |
| Q17RW2-2 | KGVPGGRGLP | 936.5505 | 10 | 0.000549984 | 0.000276589 | -1.988455417 | 0.015700976 |
| O95810 | VEGEIAEEAAEKA | 1344.6409 | 13 | 8.5195E-05 | 4.2749E-05 | -1.992912477 | 0.008317693 |
| P68032 | NNVLSGGT | 760.3715 | 8 | 8.34483E-05 | 4.17794E-05 | -1.997353934 | 0.029011306 |
| A6NK59-3 | IDEDFDTQ | 981.3927 | 8 | 0.000471427 | 0.00023361 | -2.018004711 | 0.029471203 |
| P78527-2 | VKGAAGRTDLLRLRRR | 1837.1234 | 16 | 8.81223E-05 | 4.27979E-05 | -2.059035472 | 0.018246357 |
| A0A087WV96 | KVFPFL | 749.4476 | 6 | 7.45587E-05 | 3.5695E-05 | -2.088774245 | 0.044869532 |
| Q14624 | GLPGPPDVPDHAAYHPF | 1785.8474 | 17 | 0.000194788 | 9.25313E-05 | -2.105104051 | 0.138199745 |
| Q5SR47 | AGLLGVFLALVA | 1142.7063 | 12 | 0.000107164 | 5.05773E-05 | -2.118815836 | 0.021111642 |
| O60840-2 | EPSPANGAGPG | 952.425 | 11 | 0.001071167 | 0.000505172 | -2.120401983 | 0.138104941 |
| Q9Y5A7 | SPSDSAGTSSAS | 1052.4258 | 12 | 6.46358E-05 | 3.04485E-05 | -2.122791991 | 0.120267234 |
| F5GZ06 | LLARLLVISMPASLGELRGAGAIGLLKILPEI | 3296.9985 | 32 | 0.000135401 | 6.32686E-05 | -2.140098667 | 0.072672817 |
| Q9UP95-6 | IAKDN | 559.2966 | 5 | 7.45587E-05 | 3.46765E-05 | -2.150121846 | 0.144288132 |
| Q86VD1-2 | RLIKMHEKVGSQLKLKSLLGAGVVGIVNIP | 3196.9209 | 30 | 0.000178006 | 8.27208E-05 | -2.151883756 | 0.141254045 |
| O95425-4 | HECDEGSEP | 1001.3396 | 9 | 0.000142988 | 6.61435E-05 | -2.161787391 | 0.022333374 |
| P12111-2 | YRAAEGIPKLLVLI | 1554.9497 | 14 | 0.000110881 | 5.09192E-05 | -2.177589769 | 0.054456455 |
| Q14624 | LSSRQLGLPGPPDVPDHAAYHPF | 2499.2783 | 23 | 0.000514537 | 0.000235638 | -2.183584754 | 0.09412505 |
| Q6S8J3 | VLDNKKRTALIKAVQ | 1696.0359 | 15 | 8.59382E-05 | 3.92464E-05 | -2.189706949 | 0.013131101 |
| Q9UPU3 | RVIGGIAT | 785.4759 | 8 | 4.86588E-05 | 2.13989E-05 | -2.273888475 | 0.006405551 |
| P39060 | PPGIGYEGRQGPPG | 1380.6786 | 14 | 3.30883E-05 | 1.45474E-05 | -2.274508662 | 0.069851051 |
| O95398 | WKGSVNVVTHGKGLVT | 1680.9312 | 16 | 0.000156686 | 6.7239E-05 | -2.330287564 | 0.06726372 |
| Q6ZMW3 | RLVAKAH | 793.4922 | 7 | 6.53635E-05 | 2.75736E-05 | -2.370508128 | 0.051094574 |
| Q12802-4 | APAASSLDGNKPAESSLAFSNEET | 2392.103 | 24 | 0.000364768 | 0.000153763 | -2.372271632 | 0.115496756 |
| P02671-2 | GEGDFLAEGGGVR | 1262.5891 | 13 | 0.000209673 | 8.6975E-05 | -2.410733799 | 0.083634759 |
| P51991-2 | GYGSGGGSGGYG | 974.3729 | 12 | 0.000143276 | 5.86084E-05 | -2.444622472 | 0.095680474 |
| Q76MJ5 | VSKALVHTGVAL | 1193.7131 | 12 | 0.000200459 | 8.17024E-05 | -2.453524043 | 0.038521477 |
| P60709 | DDAPRAVFPSIVG | 1342.6881 | 13 | 0.000271415 | 0.000109373 | -2.48154042 | 0.100096298 |
| A6NHT5 | AAPGAAGASVGAAAAT | 1212.6099 | 16 | 0.000129999 | 5.09192E-05 | -2.553050066 | 0.041734892 |
| O75445 | HLIPFT | 726.4064 | 6 | 0.000193638 | 7.52833E-05 | -2.572130628 | 0.132205693 |
| Q9NRC6 | ETLLRRHVRLERVLR | 1945.1809 | 15 | 6.01146E-05 | 2.33456E-05 | -2.574987513 | 0.077964208 |
| A0A0G2JMJ5 | GLKVIMGILLPPT | 1350.8308 | 13 | 0.00010636 | 4.06839E-05 | -2.614303836 | 0.023478664 |
| Q8TF62 | ELVKKYRNAVTLA | 1503.8773 | 13 | 0.000115057 | 4.26305E-05 | -2.698930943 | 0.102374394 |
| Q8TDX9-2 | SLNQAIRKEF | 1204.6564 | 10 | 6.23752E-05 | 2.28364E-05 | -2.731398171 | 0.085926475 |
| Q86XX4 | QDGTE | 548.2078 | 5 | 6.4483E-05 | 2.33456E-05 | -2.762103896 | 0.141442609 |
| Q16719 | GIRVAPVP | 807.4966 | 8 | 9.73176E-05 | 3.46765E-05 | -2.806440561 | 0.034519757 |
| P08217 | KTSMICAGGD | 981.426 | 10 | 0.000811934 | 0.000285384 | -2.845063233 | 0.061021448 |
| A6BM72 | GWTGLFCTQ | 1011.4484 | 9 | 0.00030701 | 0.000100391 | -3.058146462 | 0.105522252 |
| Q9Y5S2 | LPLIKAILTAAIVDADRIAVGLE | 2374.4199 | 23 | 5.09194E-05 | 1.62427E-05 | -3.134908058 | 0.144970532 |
| Q7Z7G8-2 | KLGSEPVVITI | 1154.691 | 11 | 0.000134673 | 4.27979E-05 | -3.14673086 | 0.056057955 |
| Q9ULI4 | AAAGRAGEAAGGPLC | 1270.6088 | 15 | 0.000120076 | 3.75514E-05 | -3.197654973 | 0.0552964 |
| Q8N2C7-7 | QQPLGRKRGLRQLRRPLLSRQK | 2683.6421 | 22 | 8.885E-05 | 2.75736E-05 | -3.222282165 | 0.139793663 |
| Q96JH7 | QTPSSLASAAAS | 1089.5302 | 12 | 0.002751596 | 0.000842678 | -3.265299053 | 0.118326839 |
| F8W9J4 | KDSPLNDMIQSNDL | 1588.7402 | 14 | 0.000147778 | 4.46543E-05 | -3.309388077 | 0.120501721 |
| Q13444-2 | NSCPCPGPAPAKT | 1241.5532 | 13 | 0.000403502 | 0.00012111 | -3.331689292 | 0.025990902 |
| Q9NYU1 | NGRFLGPLDED | 1231.5833 | 11 | 0.000326224 | 9.78549E-05 | -3.333756748 | 0.114883227 |
| Q8IXI1 | VVGAAVAA | 656.3857 | 8 | 5.09194E-05 | 1.52243E-05 | -3.344623493 | 0.084630217 |
| Q9BZF3-5 | KRVTRRWR | 1156.7053 | 8 | 6.53635E-05 | 1.94523E-05 | -3.360199247 | 0.039394723 |
| Q9C093-2 | PKGKSSGGK | 844.4766 | 9 | 9.29492E-05 | 2.75736E-05 | -3.37094821 | 0.123923548 |
| Q9H1H9-3 | KGKSKFV | 792.4857 | 7 | 6.11644E-05 | 1.78114E-05 | -3.434005427 | 0.08784259 |
| Q14624 | QLGLPGPPDVPDHAAYHPF | 2009.9635 | 19 | 6.00382E-05 | 1.57335E-05 | -3.815952874 | 0.024563984 |
| Q96JI7-3 | IMHGQ | 584.274 | 5 | 0.000128391 | 3.24854E-05 | -3.952271868 | 0.103865889 |
| A8MVG2 | ILAVLLFF | 934.5891 | 8 | 0.000101264 | 2.33456E-05 | -4.337604002 | 0.057062146 |
| P0C0L4-2 | TKDDPDAPLQPVTPLQLFEGR | 2336.2012 | 21 | 6.9157E-05 | 1.52243E-05 | -4.542557435 | 0.004766376 |
| Q9ULT6-2 | QGTHSLGSWGG | 1085.489 | 11 | 0.001178913 | 0.000249904 | -4.717467044 | 0.145333743 |
| P98164 | ICSCTAGFETNVFDRTSCL | 2065.8906 | 19 | 0.000160767 | 3.4E-05 | -4.728454071 | 0.040302101 |
| Q8WY21 | PGQHILVAVLPGL | 1312.7866 | 13 | 4.63982E-05 | 8.12135E-06 | -5.713111021 | 0.055024818 |
